# Supplementary figures and images for: Suppressing neutrophil itaconate production attenuates Mycoplasma pneumoniae pneumonia
Source: PLoS Pathog. 2024 Nov 5;20(11):e1012614. doi: 10.1371/journal.ppat.1012614 (PMC11567624; doi:10.1371/journal.ppat.1012614)

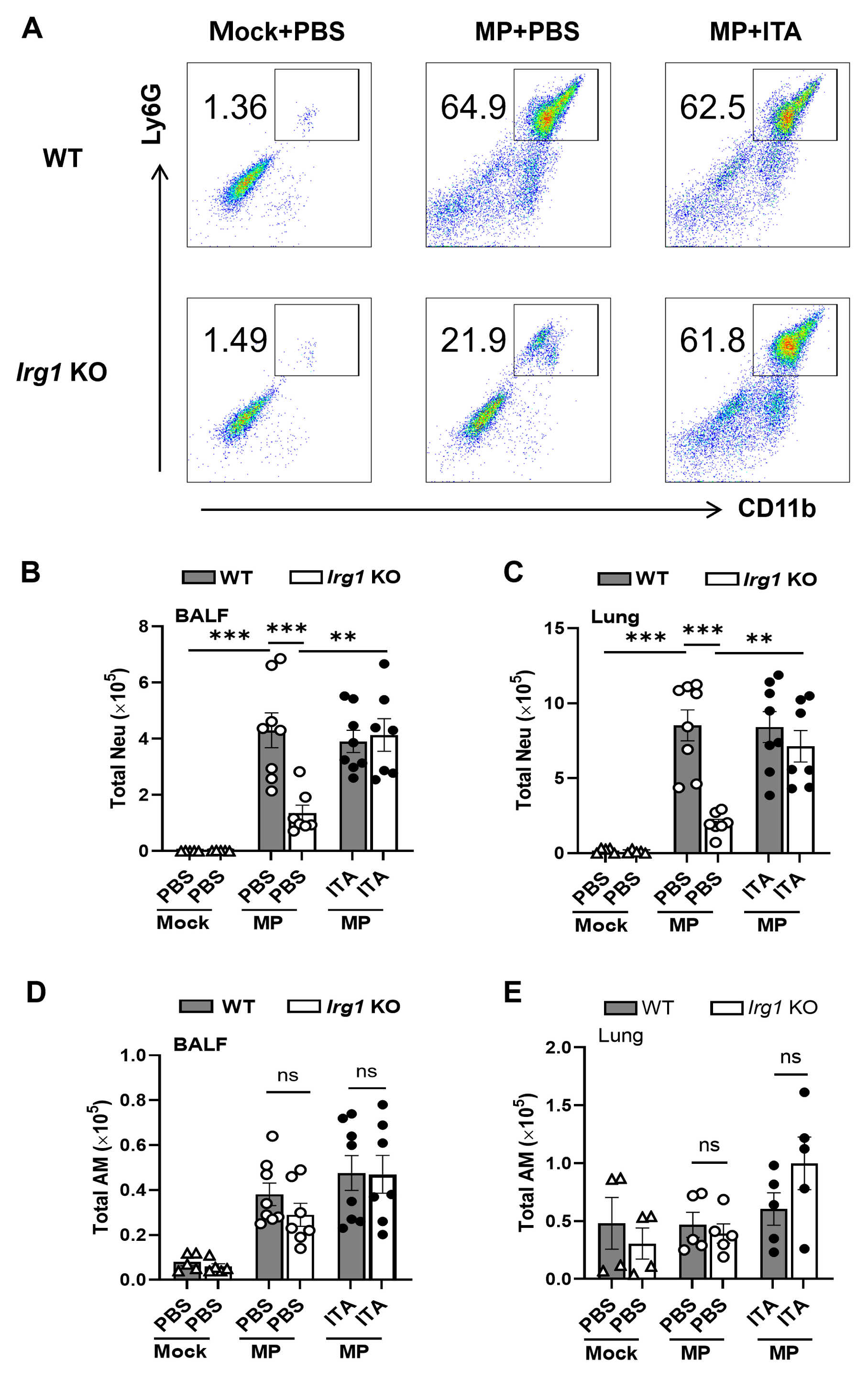

Supplement: S1 Fig — The experiment was described as in Fig 1D–1J. (A) Representative flow cytometric analysis of CD11b+Ly6G+ neutrophils gated on CD45+ cells in BALF from WT and Irg1 KO mice. (B) Neutrophil numbers in BALF (n = 5–8), pooled from three independent experiments. (C) Neutrophil numbers in lung (n = 5–8), pooled from three independent experiments. (D) Alveolar macrophages (AM) numbers in BALF (n = 5–8), (E) AM numbers in lung (n = 4–5), pooled from three independent experiments. Data are presented as mean ± SEM. Statistical significance tested by one-way ANOVA test (*, p<0.05; **, p<0.01; ***, p<0.001). (TIF) [file ppat.1012614.s001.tif]

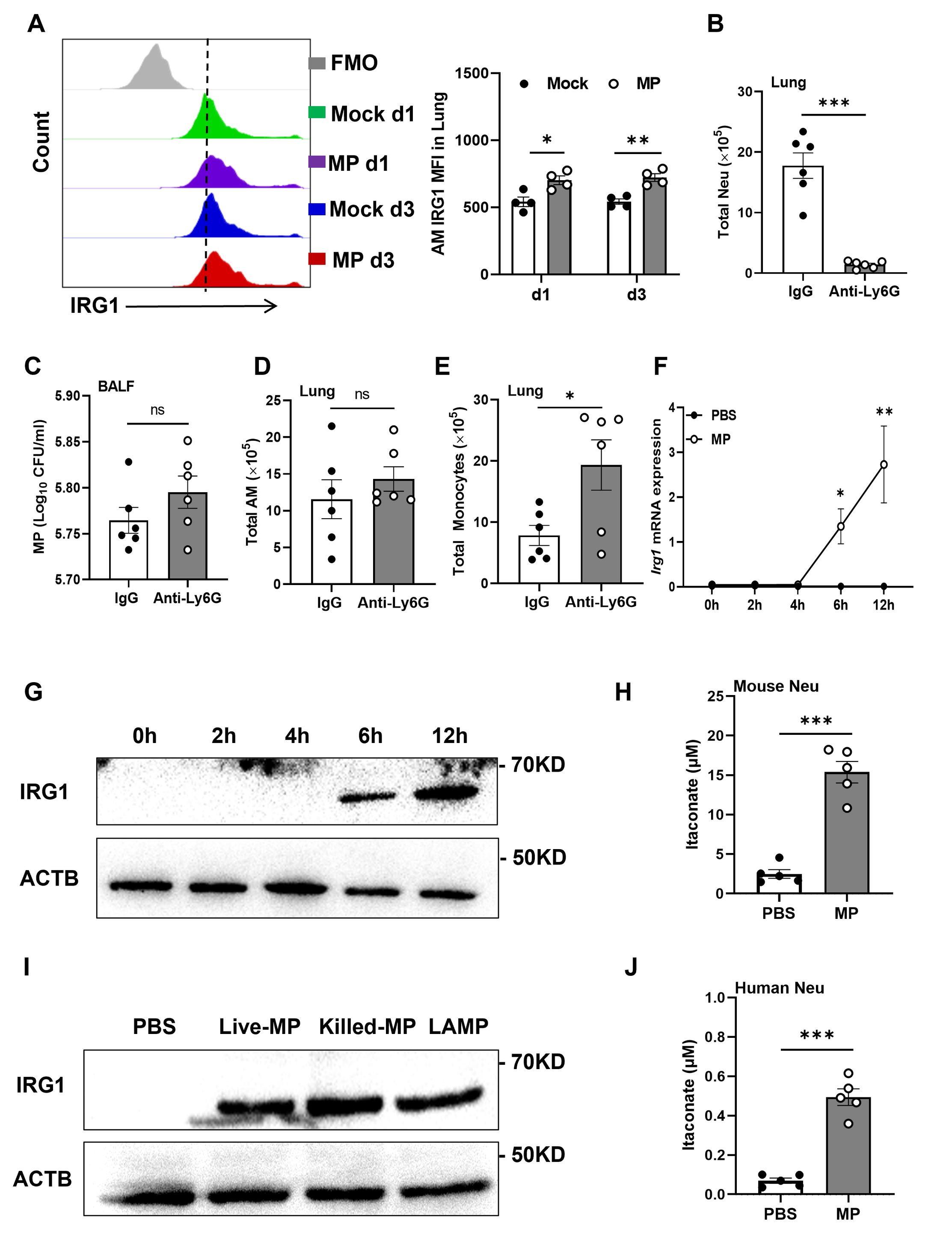

Supplement: S2 Fig — Flow cytometric analysis of IRG1 expression in lung alveolar macrophages (AM) at day 1 and day 3 after M. pneumoniae (MP) infection in mice (n = 4 per group). (B-E) The experiment was described as in Fig 2E–2G (n = 6 per group). (B) Neutrophil numbers in lung. (C) M. pneumoniae load in BALF. (D) Alveolar macrophages (AM) numbers in lung. (E) Monocyte numbers in lung. (F and G) Dynamic expression of Irg1 mRNA expression (F) and IRG1 protein expression (G) in mouse BM neutrophils after M. pneumoniae (MOI = 0 or 10) infection in vitro. The experiment was performed three times independently. (H) Itaconate concentrations in the culture supernatants of mouse neutrophils after M. pneumoniae infection for 20 h. (I) Mouse BM neutrophils were treated with live M. pneumoniae, heat (96°C for 10 minutes)-killed M. pneumoniae and 5 μg/ml M. pneumoniae lipid-associated membrane proteins (LAMP) for 12 h followed by western blot analysis of the IRG1 protein, representative of three experiments. (J) Itaconate concentrations in the culture supernatants of human neutrophils after M. pneumoniae infection for 20 h. Data are presented as mean ± SEM. Statistical significance tested by unpaired, two-tailed Student’s t test (*, p<0.05; **, p<0.01; ***, p<0.001). (TIF) [file ppat.1012614.s002.tif]

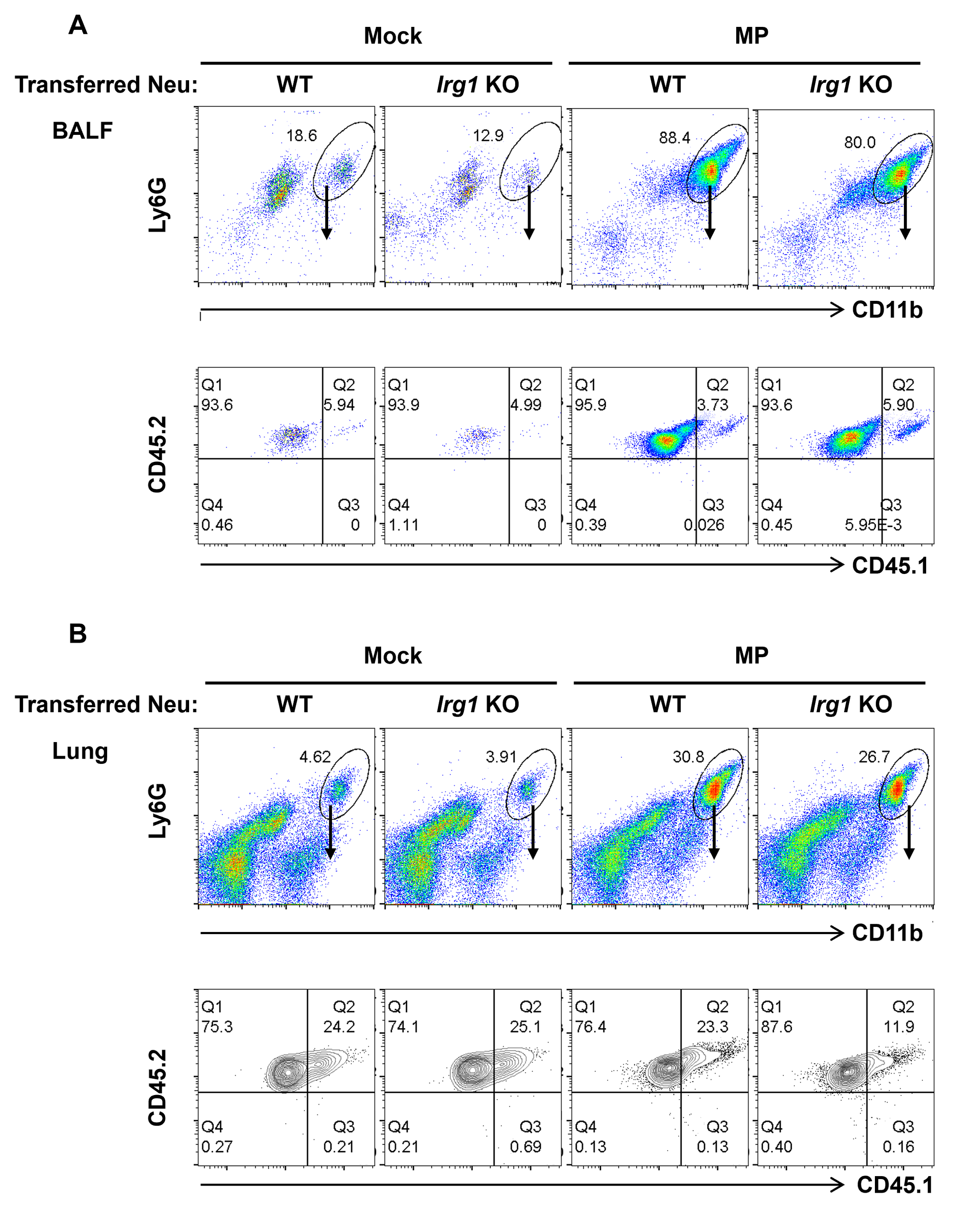

Supplement: S3 Fig — Sorted BM neutrophils (CD45.2 background) from WT and Irg1 KO mice were intravenously transferred into CD45.1/2 mice, respectively. Then, the recipient mice were i.n. infected by M. pneumoniae (MP) or treated with PPLO medium (Mock group). Mice were sacrificed at day 1 after infection to analyze transferred neutrophils (CD45.1-CD45.2+ cells) and non-transferred neutrophils (CD45.1+CD45.2+ cells) in BALF (A) and lung (B) by flow cytometry. (TIF) [file ppat.1012614.s003.tif]

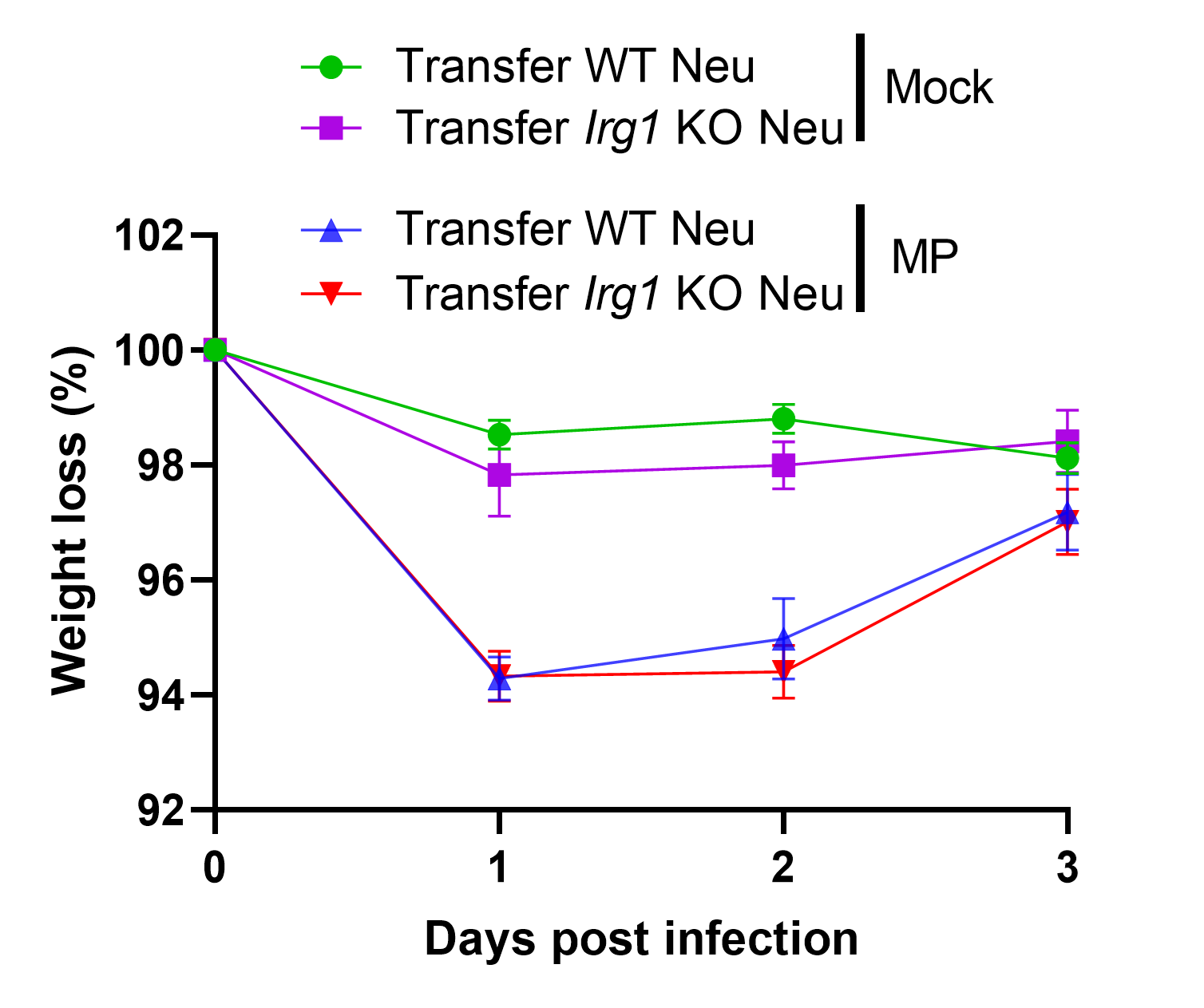

Supplement: S4 Fig — The experiment was described as in Fig 3. Data are pooled from two independent experiment and are presented as mean ± SEM. (TIF) [file ppat.1012614.s004.tif]

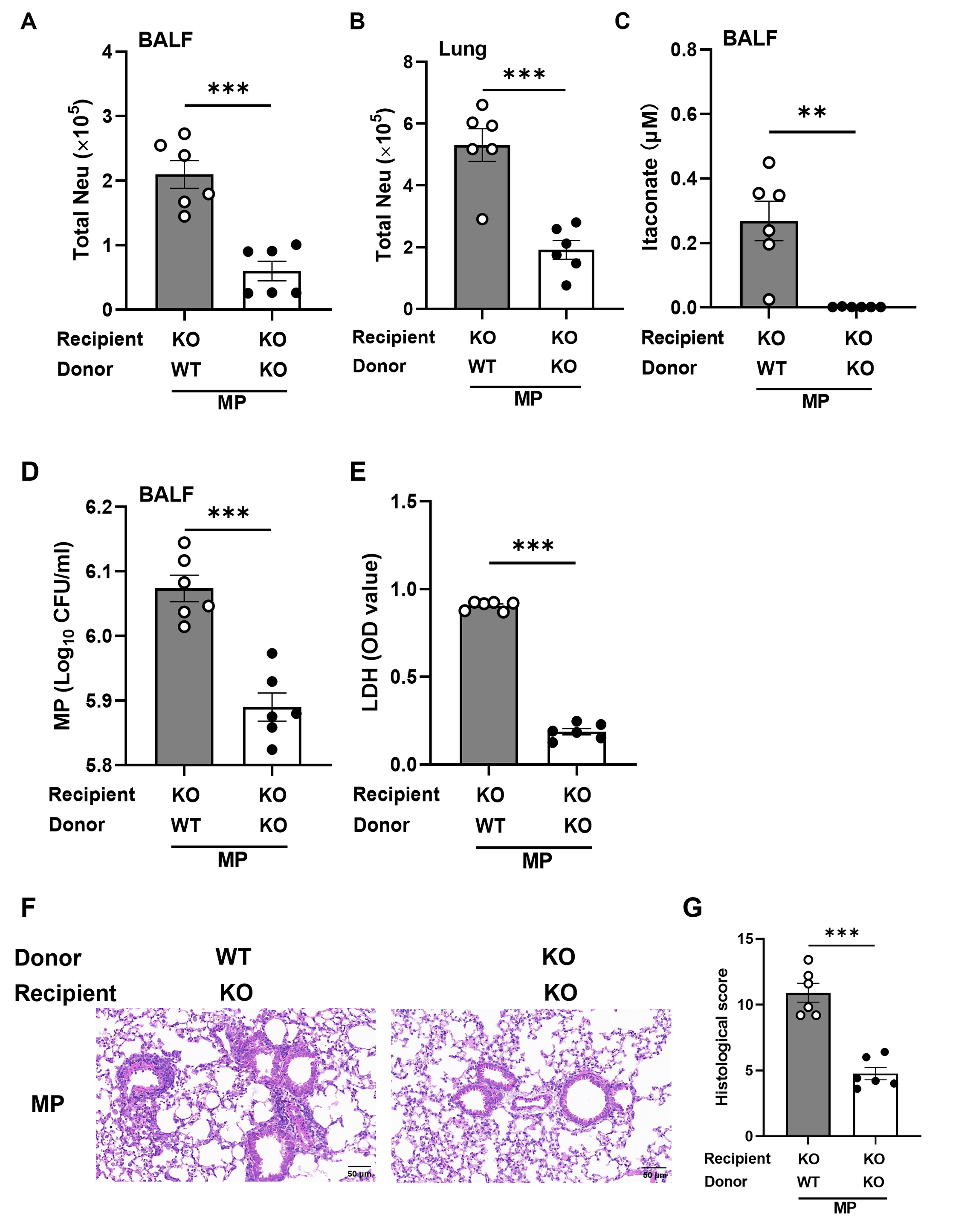

Supplement: S5 Fig — (A-E) Sorted BM neutrophils from WT and Irg1 KO mice were intravenously transferred into Irg1 KO mice followed by M. pneumoniae (MP) infection. Mice were sacrificed at day 3 after M. pneumoniae infection (n = 6 per group). (A) Neutrophil numbers in BALF. (B) Neutrophil numbers in lung. (C) Itaconate concentrations in BALF. (D) M. pneumoniae load in BALF. (E) LDH levels in BALF. (F) Representative H&E staining of lung tissue. Scale bar, 50 μm. (G) Histological scores. Data are pooled from two independent experiment and are presented as mean ± SEM. Statistical significance tested by unpaired, two-tailed Student’s t test (*, p<0.05; **, p<0.01; ***, p<0.001). (TIF) [file ppat.1012614.s005.tif]

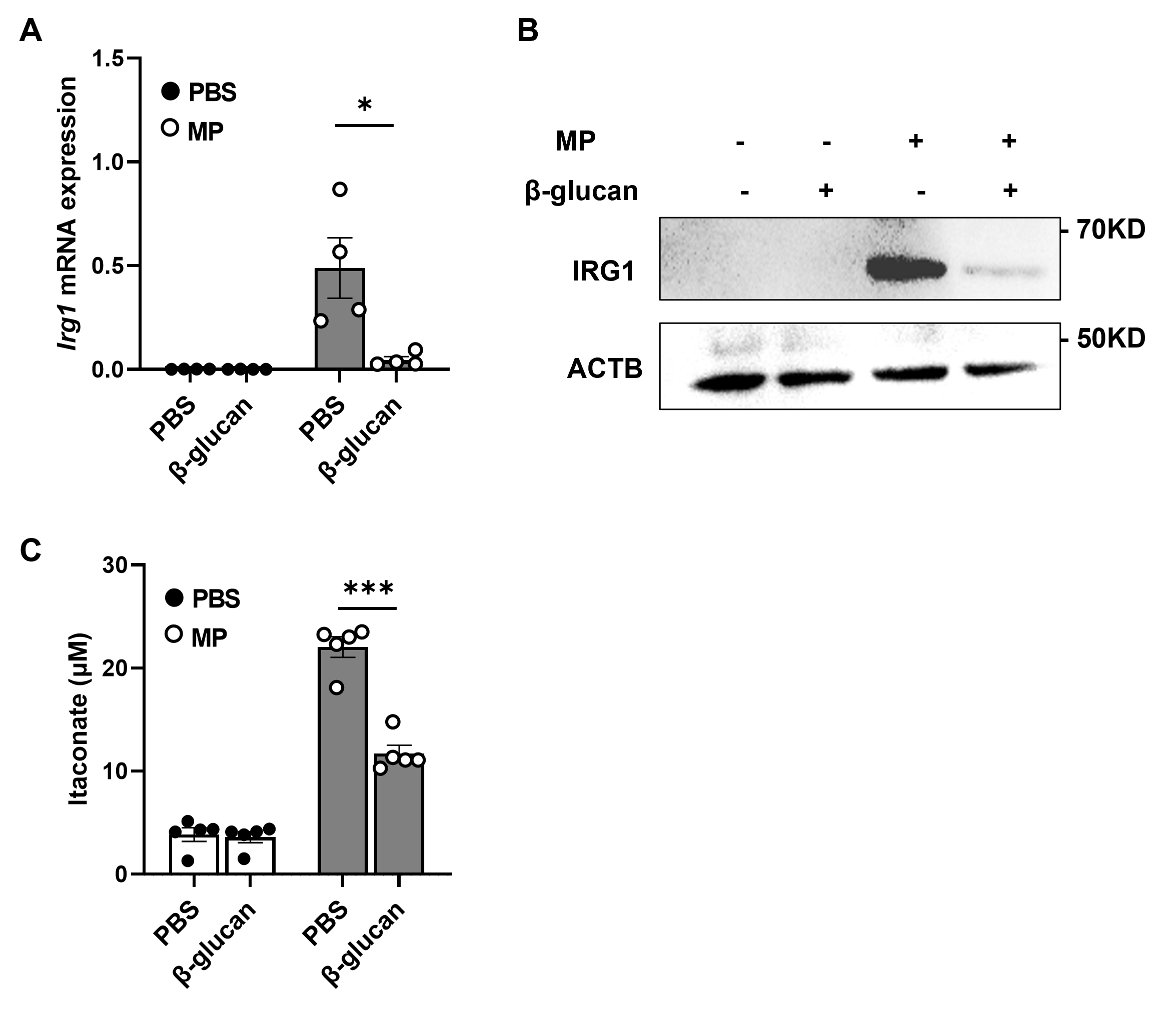

Supplement: S6 Fig — (A and B) Mouse BM neutrophils were pretreated with β-glucan (0 or 5 μg/ml) for 1 h followed by M. pneumoniae (MP) infection for 12 h. The experiment was performed three times independently. (A) The Irg1 mRNA expression in neutrophils. (B) IRG1 protein expression in neutrophils, representative of three experiments. (C) Itaconate concentrations in the culture supernatants. Data are presented as mean ± SEM. Statistical significance tested by unpaired, two-tailed Student’s t test (*, p<0.05; **, p<0.01; ***, p<0.001). (TIF) [file ppat.1012614.s006.tif]

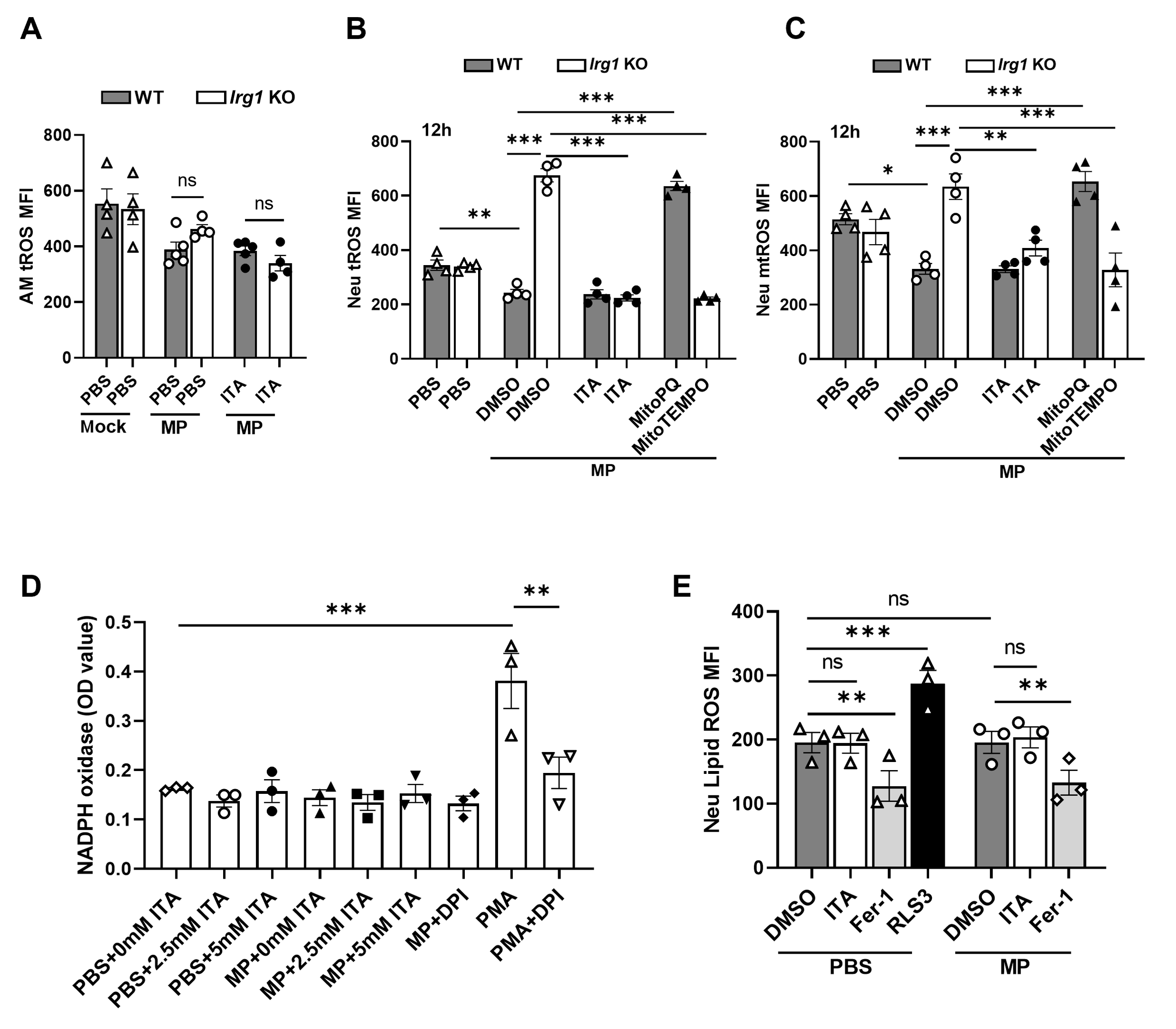

Supplement: S7 Fig — (A) Flow cytometric analysis of the total ROS of lung (alveolar macrophages) AM from WT and Irg1 KO mice at day 3 after M. pneumoniae (MP) infection with or without exogenous itaconate (ITA) treatment (n = 4 or5 per group). Data are presented as mean ± SEM. Statistical significance tested by unpaired, two-tailed Student’s t test. (B and C) Mouse BM neutrophils from WT and Irg1 KO mice were pretreated with 5 mM itaconate, 1 μM MitoPQ, or 100 μM MitoTEMPO for 1 h followed by the infection with M. pneumoniae for 12 h. Total ROS (B) and mtROS (C) production were determined by flow cytometry. (D) Neutrophil NADPH oxidase activity in neutrophils after 2 h infection with M. pneumoniae (MOI = 0 or 10) in the presence of itaconate (0–5 mM), 10 μM NADPH oxidase inhibitor DPI, 1 mM NADPH oxidase activator PMA. (E) Neutrophil lipid ROS production in neutrophils after 2 h infection with M. pneumoniae (MOI = 0 or 10) in the presence of 5 mM itaconate, 10 μM ferroptosis inhibitor ferrostatin-1 (Fer-1), 10 μM ferroptosis activator (1S,3R)-RSL3 (RSL3). Data are pooled from three independent experiments and are presented as mean ± SEM. (B-E) Statistical significance tested by one-way ANOVA test (*, p<0.05; **, p<0.01; ***, p<0.001). (TIF) [file ppat.1012614.s007.tif]

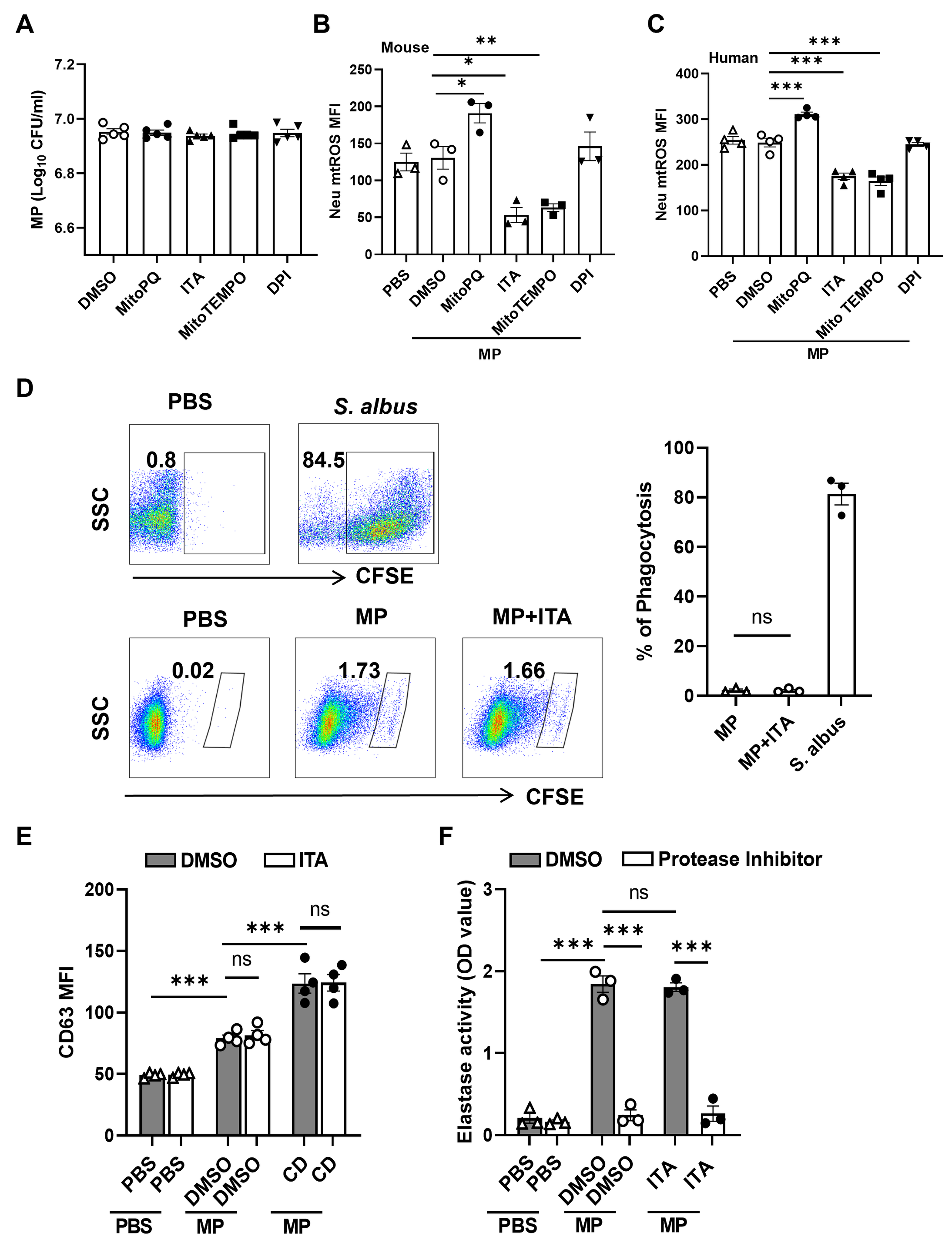

Supplement: S8 Fig — (A) M. pneumoniae (MP) detection after 5 mM itaconate (ITA), 1 μM MitoPQ, 100 μM MitoTEMPO, or 10 μM DPI treatment for 2 h. (B) Mouse BM neutrophils and (C) human neutrophils were pretreated with 5 mM itaconate, 1 μM MitoPQ, 100 μM MitoTEMPO, 10 μM DPI for 1 h followed by M. pneumoniae infection for 2 h, and neutrophils mtROS were detected by flow cytometry. (D) Bacterial phagocytosis by neutrophils. Staphylococcus albus (S. albus) was used as a positive control. (E and F) Neutrophils in the presence of 5 mM itaconate for 1 h followed by M. pneumoniae infection for 2 h. (E) cells were stained with anti-CD63 antibodies and detected by flow cytometry for neutrophil degranulation, (F) and neutrophil elastase activity was measured with OD 400 nm. Data are pooled from three independent experiments and are presented as mean ± SEM. Statistical significance tested by one-way ANOVA test (*, p<0.05; **, p<0.01; ***, p<0.001). (TIF) [file ppat.1012614.s008.tif]

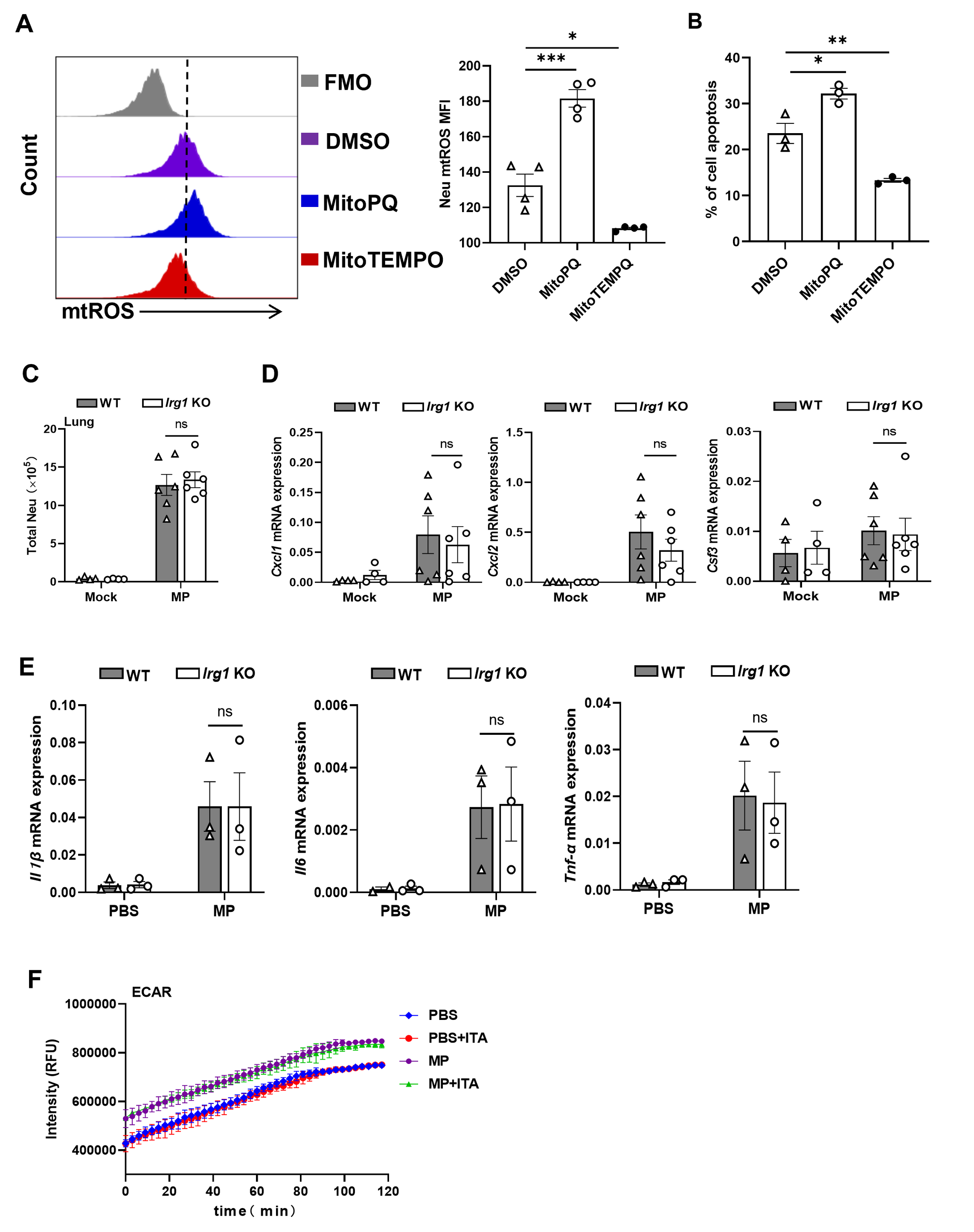

Supplement: S9 Fig — (A and B) Mouse BM neutrophils were treated with 1 μM MitoPQ or 100 μM MitoTEMPO in vitro for 12 h. Neutrophil mtROS production (A) and the percentage of apoptotic cells (B) were pooled from three independent experiments. Statistical significance tested by one-way ANOVA test. (C) The number of neutrophils in the lung from WT and Irg1 KO mice at day 1 after M. pneumoniae (MP) infection (n = 4–6 per group), pooled from two independent experiments. Statistical significance tested by unpaired, two-tailed Student’s t test. (D) The mRNA expression of Cxcl1, Cxcl2 and Csf3 in the mouse neutrophils from WT and Irg1 KO mice at day 1 after M. pneumoniae infection (n = 4–6 per group), pooled from two independent experiments. Statistical significance tested by unpaired, two-tailed Student’s t test. (E) The mRNA expression of Il-1β, Il-6, and Tnf-α in the mouse neutrophils of WT and Irg1 KO mice after 12 h of infection with M. pneumoniae, pooled from three independent experiments. Data are presented as mean ± SEM. Statistical significance tested by unpaired, two-tailed Student’s t test. (F) Neutrophil extracellular acidification rate (ECAR) after 2 h infection with M. pneumoniae (MOI = 0 or 10) in the presence of itaconate (ITA, 0 or 5 mM), pooled from three independent experiments. RFU: relative fluorescence units. *, p<0.05; **, p<0.01; ***, p<0.001. (TIF) [file ppat.1012614.s009.tif]
