# Supplementary material for: Suppressing neutrophil itaconate production attenuates Mycoplasma pneumoniae pneumonia
Source: PLoS Pathog. 2024 Nov 5;20(11):e1012614. doi: 10.1371/journal.ppat.1012614 (PMC11567624; doi:10.1371/journal.ppat.1012614)
Supplement: S1 Table — (DOCX) [file ppat.1012614.s010.docx]

S1 Table. Demographic and clinical information of patients with severe *M. pneumoniae* pneumonia.

| Parameters | ^a^ Patients, n=17 | ^b^ Patients, n=4 |
| --- | --- | --- |
| Age (mean ± SD） | 6.05 ± 2.03 | 5.13 ± 1.92 |
| Male (n, %) | 7 (41.18) | 3（75） |
| Female (n, %) | 10 (58.82) | 1（25） |
| Temperature (mean ± SD, ℃） | 39.43 ± 0.79 | 39.1 ± 0.18 |
| Respiratory rate (mean ± SD） | 24.88 ± 2.71 | 25.25 ± 0.96 |
| Day of cough (mean ± SD） | 6.56 ± 3.46 | 3.5 ± 0.58 |
| Day of fever (mean ± SD） | 6.44 ± 3.44 | 3.75 ± 0.96 |
| CRP (mean ± SD, mg/L） | 24.51 ± 40.26 | ND |
| HB (mean ± SD, g/L） | 119.76 ± 7.38 | 114 ± 2.83 |
| LDH (mean ± SD, U/L） | 309.48 ± 111.52 | ND |
| WBC (mean ± SD, x 109/L） | 7.82 ± 3.56 | 6.22 ± 1.91 |
| Blood neutrophils (mean ± SD, %） | 64.96 ± 10.63 | 42.6 ± 23.99 |
| Blood neutrophils (mean ± SD, x 109/L） | 1.38 ± 0.73 | 2.55 ± 0.92 |
| Blood lymphocyte (mean ± SD, %） | 28.42 ± 14.43 | 46.95 ± 18.75 |
| Blood platelet count (mean ± SD, %） | 275.53 ± 86.95 | 443.5 ± 228.28 |
| Chest CT findings |  |  |
| Consolidation (n, %) | 7 (41.17) | 1 (25) |
| Bronchovascular bundles thickening (n, %) | 12 (70.58) | 0 |
| Patchy high-density shadow (n, %) | 17 (100) | 4 (100) |
| Linear opacities (n, %) | 5 (29.41) | 0 |
| Nodules (n, %) | 2 (11.76) | 0 |
| Lymphadenopathy (n, %) | 1 (5.88) | 0 |
| Pleural effusion (n) | 0 | 0 |
| Chest imaging score |  |  |
| 1 (n, %) | 10 (58.82) | 1 (25) |
| 2 (n, %) | 7 (41.18) | 3 (75) |

^a^ BALF samples were used for measuring itaconate concentrations, corresponding to Fig 2J.

^b^ BALF samples were used for determining IRG1 expression in neutrophils and non-neutrophils by flow cytometry, corresponding to Fig 2K.

CRP: C-reactive protein; HB: Hemoglobin; WBC: White blood cell; SD: Standard deviation; ND: not determined.
