# Supplementary material for: Suppressing neutrophil itaconate production attenuates Mycoplasma pneumoniae pneumonia
Source: PLoS Pathog. 2024 Nov 5;20(11):e1012614. doi: 10.1371/journal.ppat.1012614 (PMC11567624; doi:10.1371/journal.ppat.1012614)

(1) Gating strategy for lung neutrophils in mice

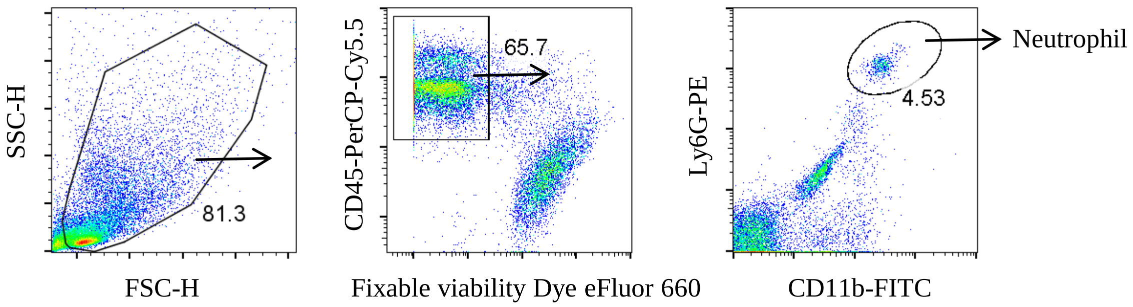

(2) Gating strategy for lung alveolar macrophages in mice

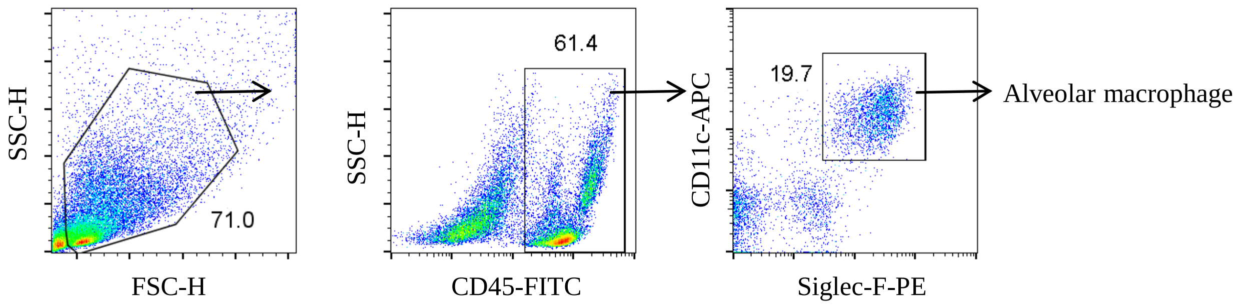

(3) Gating strategy for lung monocytes and neutrophils in mice

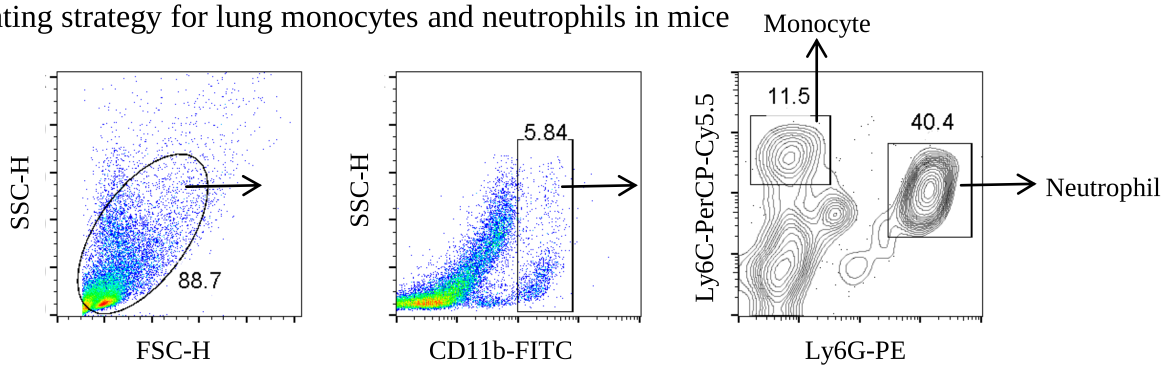

(4) Gating strategy for human neutrophils in BALF from patients with *M. pneumoniae* pneumonia

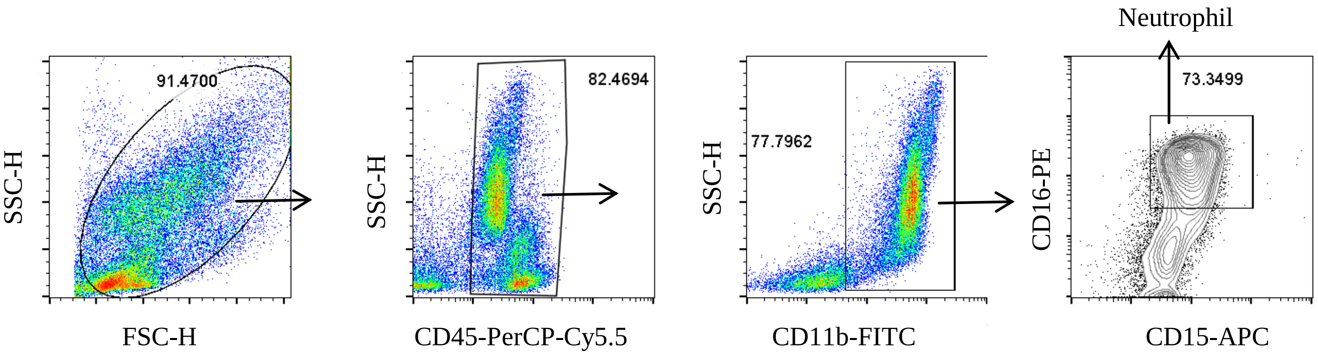

(5) The representative flow data for the purified bone marrow neutrophils in mice

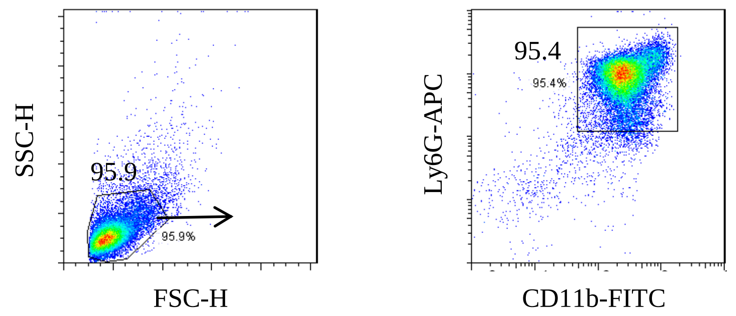

(6) The representative flow data for the purified lung neutrophils in mice

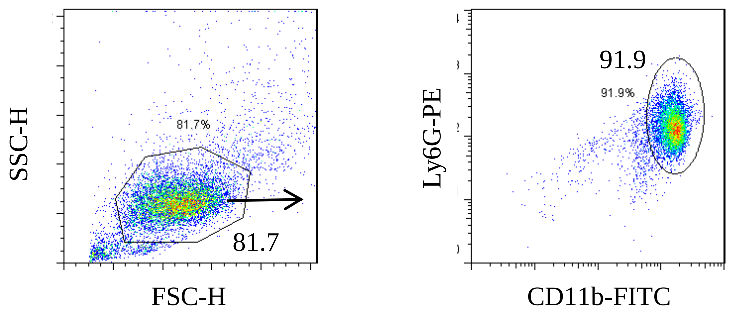

(7) The representative flow data for the purified human neutrophils from blood

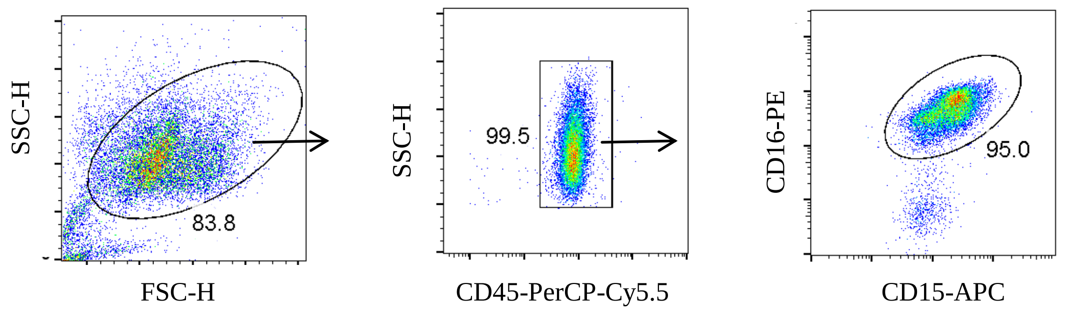

Supplement: S1 File — (PDF) [file ppat.1012614.s013.pdf]
