# Supplementary material for: Suppressing neutrophil itaconate production attenuates Mycoplasma pneumoniae pneumonia
Source: PLoS Pathog. 2024 Nov 5;20(11):e1012614. doi: 10.1371/journal.ppat.1012614 (PMC11567624; doi:10.1371/journal.ppat.1012614)
Supplement: Attachment — Submitted filename: Our response to comments.pdf [file ppat.1012614.s014.pdf]

PPATHOGENS-D-24-00902

Response to Editors and Reviewers

Dear Editors and Reviewers,

We sincerely thank the editors and all the reviewers for the valuable comments on our manuscript. Following the comments, we have made extensive modifications to our manuscript and supplemented extra data to make our results convincing (changes are highlight in red). Hopefully, you will find our revised manuscript suitable for publication at *PLOS Pathogens*. Our point-to-point responses to the editors' and reviewers' comments are described below:

Response to the Editors

All reviewers found the manuscript to be interesting, well-written, and potentially valuable. However, two of the reviewers identified a number of significant shortcomings in terms of description of important experimental details, and reviewer #3 identified at least two experiments (associated with Figures 3 and 4) that would make the findings a lot clearer. Finally, given that *Mycoplasma pneumoniae* is a human pathogen but all the findings are in a mouse model, it is important to add some discussion of how these findings relate to both human disease and any other studies involving human neutrophils. Please address all the reviewers' major and minor comments in a revised version of this manuscript.

Response: We greatly appreciate your valuable comments to our work. We have provided extra data, especially regarding Fig 3 and Fig 4, to make our findings convincing. In addition, we have added some discussion of how our findings relate to both human disease and any other studies involving human neutrophils as: Neutrophil accumulation in the lungs is a hallmark feature of *M. pneumoniae* pneumonia in children [6, 7, 10, 13]. Importantly, we observed a positive correlation between neutrophil counts and itaconate concentrations in the BALF from patients with severe *M. pneumoniae* pneumonia. Meanwhile, neutrophils had a higher level of IRG1 expression than non-neutrophils in BALF from severe patients with *M. pneumoniae*

infection, suggesting that neutrophils may also be the main source of itaconate during *M. pneumoniae* infection in humans. Notably, the concentration of itaconate measured in the BALF of human patient with *M. pneumoniae* pneumonia was much lower than that in infected mice (20 nM vs. 1000 nM). Meanwhile, mouse neutrophils produced more itaconate than human neutrophils after *M. pneumoniae* infection *in vitro*. This apparent lower capacity of human IRG1 to produce itaconate is in line with earlier reports for human macrophages[54, 55], and may be due to the reduced enzymatic activity as described previously[56]. Despite this discrepancy, itaconate impaired human neutrophil bacterial killing by inhibiting mtROS production upon *M. pneumoniae* infection, similar to the results in mouse neutrophils. These results suggest that inhibition of human neutrophil itaconate production may exert a protective effect in *M. pneumoniae*-infected patients.

Our manuscript has been carefully modified and improved according to your and reviewers' suggestions as described below.

Responses to Reviewer's Comments

Part I – Summary

Please use this section to discuss strengths/weaknesses of study, novelty/significance, general execution and scholarship.

Reviewer #1: Wang and colleagues report on the role of ACOD1/Irg1 and itaconate in a mouse model of *Mycoplasma pneumoniae* infection. While itaconate production by macrophages has been in the focus of several studies on bacterial infection in the mouse model, this study focusses on Acod1 expression and itaconate production by neutrophils. Further, while itaconate production has been shown to be protective in other bacterial infection models due to its antimicrobial and/or immunoregulatory activity, in the *M. pneumoniae* model reported here, the opposite effect is observed with reduced microbial burden, cytokine production and tissue damage in *Acod1*$^{-/-}$ mice. These results are novel and interesting, as they suggest that itaconate has proinflammatory activity in certain infections. The authors provide evidence that

interventions to reduce itaconate production during infection (e.g. transfer of *Acod1*$^{-/-}$ neutrophils, treatment with β-glucan) have a beneficial effect in the mouse infection model. They also show that itaconate inhibits mitochondrial ROS production by neutrophils and link this to reduced killing of *M. pneumoniae*. The study is well designed and experiments are well performed, the manuscript is written very well and the sequence of results are easy to follow. There are number of issues concerning the description and interpretation of the results that need to be addressed.

Response: Thanks for your great comments and encouragement. We have addressed the issues concerning the description and interpretation of the results as you raised in Part II and Part III.

Reviewer #2: This manuscript describes a series of studies that demonstrate that itaconate-producing neutrophil promotes *Mycoplasma pneumoniae* pneumonia in a mouse model. Overall this is a well written manuscript that clearly states the rationale for each of the studies and their results.

The strength of this manuscript is that the authors have used multiple approaches to corroborate their results, i.e., it is not one experiment but multiple approaches to confirm the results. Thus the data appear strong.

Another strength is the overall conclusions are well supported and significant. It is possible that if proven true in human *M. pneumoniae* infection, then this could be a possible target for therapy. It may also be applicable to other diseases where excessive inflammation may contribute to pathology.

One limitation is that these studies were done in a murine model of *M. pneumoniae* disease, which is not a great model for the human disease. However, it is one of the best available. So, a statement about this limitation and how results may or may not lead to understanding in human disease would be interesting and I think strengthen the manuscript.

Response: Thanks for your positive and valuable comments. We have added some discussion of how our findings relate to both human *M. pneumoniae* disease and a statement about the limitation as: Neutrophil accumulation in the lungs is a hallmark

feature of *M. pneumoniae* pneumonia in children[6, 7, 10, 13]. Importantly, we observed a positive correlation between neutrophil counts and itaconate concentrations in the BALF from patients with severe *M. pneumoniae* pneumonia. Meanwhile, neutrophils had a higher level of IRG1 expression than non-neutrophils in BALF from severe patients with *M. pneumoniae* infection, suggesting that neutrophils may also be the main source of itaconate during *M. pneumoniae* in humans. Notably, the concentration of itaconate measured in the BALF of human patient with *M. pneumoniae* pneumonia was much lower than that in infected mice (20 nM vs. 1000 nM). Meanwhile, mouse neutrophils produced more itaconate than human neutrophils after *M. pneumoniae* infection *in vitro*. This apparent lower capacity of human IRG1 to produce itaconate is in line with earlier reports for human macrophages[54, 55], and may be due to the reduced enzymatic activity as described previously[56]. Despite this discrepancy, itaconate impaired human neutrophil bacterial killing by inhibiting mtROS production, similar to the results in mouse neutrophils. These results suggest that inhibition of human neutrophil itaconate production may exert a protective effect in *M. pneumoniae*-infected patients.

Reviewer #3: The paper "Suppressing neutrophil itaconate production attenuates *Mycoplasma pneumoniae* pneumonia" by Wang et al investigates the role of neutrophils and their production of itaconate during *Mycoplasma pneumoniae* pneumonia (MPP).

A major strength of the study is the use of different in-vitro and in-vivo mice models and assays (such as Irg1- KO mice, pharmacological inhibitors etc) next to the use of human and MPP-patient derived neutrophils to interrogate the source and role of itaconate during MPP.

Weaknesses: poor description of patient cohort in terms of disease severity; days after onset of symptoms is not clear. Discussion could benefit from context: what is known about itaconate-producing neutrophils in airway diseases/infections? what about other myeloid cells: do they contribute to the process?

also considering 1) the findings in patients with severe MPP (Fig2J);

and 2) the experiments with the Dectin1-ligand beta-glucan: are observed effects on Irg1 expression due to direct effects of beta-glucan on neutrophils or indirect via monocytes/macrophages?

Response: Thanks for your positive comments and good suggestions. We have added the description of patient cohort in terms of disease severity and days after onset of symptoms in S1 Table. Meanwhile, we have added more discussion about the role of itaconate-producing neutrophils in airway diseases/infections and also discussed the role of monocytes and macrophages during *M. pneumoniae* infection as: Moreover, *Irg1* deficiency in myeloid cells including monocytes/macrophages, neutrophils, and some dendritic cells, exacerbates *Mtb*-induced lung pathology and disease, suggesting a protective role of itaconate during *Mtb* infection [25]. These results and ours indicate that itaconate-producing neutrophils have different effects in different microbial pulmonary infections. Macrophages play an important role in eliminating *M. pneumoniae* [8] We also found that *M. pneumoniae* induced IRG1 expression in alveolar macrophages; however, *Irg1* deficiency did not affect alveolar macrophage ROS production. Whether itaconate could regulate monocyte/macrophage function during *M. pneumoniae* infection remains to be investigated.

As we found that β-glucan can inhibited IRG1 expression and itaconate production in neutrophils after *M. pneumoniae* infection *in vitro* (S6 Fig), β-glucan probably exerts direct effects on *Irg1* expression in neutrophils upon infection.

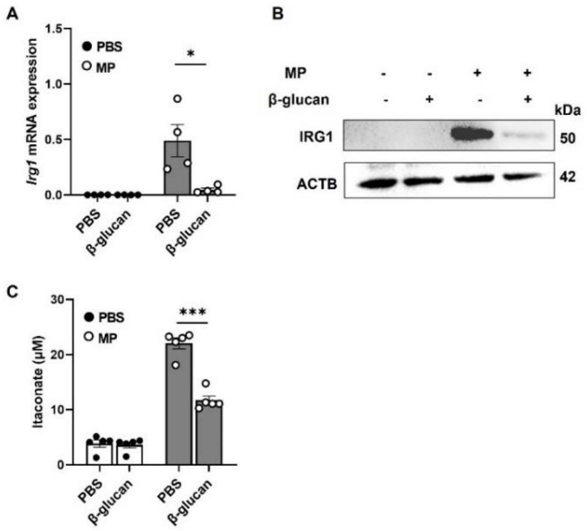

S6 Fig. β-glucan inhibits *M. pneumoniae*-induced *Irg1* expression in neutrophils *in vitro*.

Part II – Major Issues: Key Experiments Required for Acceptance

Please use this section to detail the key new experiments or modifications of existing experiments that should be absolutely required to validate study conclusions.

Reviewer #1: Specific comments:

Figure 1: mice are infected with 10e8 CFU, and when measured 3 days later, the BAL fluid contains less than 10e6 CFU/ml (Fig. 1D). Therefore, it appears that only a quite small fraction of the infecting bacteria is left after three days of infection. How many bacteria are recovered from the lung tissue at this time? Which volume was used to obtain the BALF cells?

Response: As described in Materials and methods section, BALF was obtained by flushing the lungs with 0.8 ml of cold PBS twice via a thin tube inserted into a cut made in the trachea. It is true that only a quite small fraction of the infecting bacteria is left after three days of *M. pneumoniae* infection, probably due to the elimination effects by host immune response. We determined *M. pneumoniae* load in the lung homogenates after three days of *M. pneumoniae* infection and obtained similar results as shown in Figure 1F. The bacteria burden recovered from the lung tissue were a little higher than that from BALF.

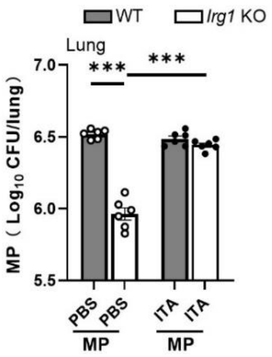

The histopathology (Fig. 1G) shows increased leukocyte infiltration in the WT mice and after administration of ITA in representative images. To be informative, quantitative data from analysis of several sections from all the mice in the experiment

are required. These could be obtained e.g. by measuring the area of air-filled alveoli vs. cellular infiltrates.

Response: To be informative, H&E images per mouse were blindly scored based on the severity of peribronchiolar/peribronchiolar infiltrates (including quantity and quality), bronchiolar/luminal exudates, perivascular infiltrates, and parenchymal pneumonia as described previously (Martin et al, 2001, Am J Respir Cell Mol Biol. PMID: 11350827; Cimolai et al, 1992, Microbiol Immunol. PMID: 1513263). Quantitative data has been supplemented in all Figures containing H&E results.

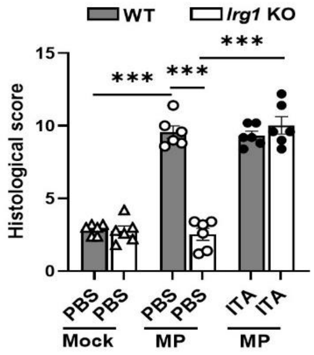

The intranasal application of 10 mg/kg itaconate increases CFU and cytokine responses in *Acod1*$^{-/-}$ mice. It would be informative to also show itaconate concentrations in the BALF of treated mice in Fig. 1C.

Response: We have supplemented itaconate concentrations in the BALF of treated mice in Fig 1D. Our results showed that the itaconate concentrations in BALF of itaconate-treated KO mice were significantly increased after three days of *M. pneumoniae* infection.

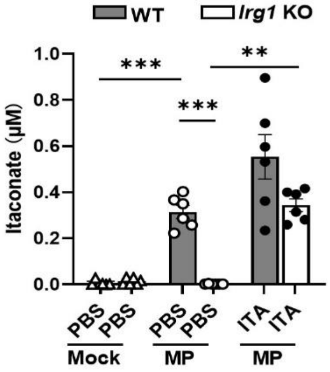

Figure 2: the data convincingly demonstrate that itaconate production in the lungs of *M. pneumoniae*-infected mice is primarily derived from neutrophils. For human patients with severe pneumonia, expression of IRG1 protein in neutrophils is also shown by Western blot and intracellular staining. It is remarkable that the concentration of itaconate measured in the BALF of human patient is much lower than in mice (20 nM vs. 1000 nM). This apparent lower capacity of human ACOD1 to produce itaconate would be in line with earlier reports for human macrophages (Michelucci 2013 PNAS, Kohl 2023 EMBO Mol Med) and could be due to the reduced enzymatic activity reported previously (Chen…Pessler 2019 PNAS). This difference between species should be discussed. In addition, the content of itaconate in lysates and culture supernatants of mouse vs. human neutrophils should be analyzed to validate production of itaconate in humans in comparison with the murine system.

Response: Thanks for the good suggestions. It is true that the itaconate concentrations in the BALF of human patient with *M. pneumoniae* pneumonia were much lower than that in mice. As you suggested, we analyzed itaconate content in culture supernatants of mouse vs. human neutrophils after 20 h of *M. pneumoniae* infection (S2H and S2J Fig). Indeed, *M. pneumoniae* infection remarkably induced itaconate production in both mouse and human neutrophils, and mouse neutrophils produced more itaconate than human neutrophils. We have discussed this difference between species in the Discussion part as: Notably, the concentration of itaconate measured in the BALF of human patient with *M. pneumoniae* pneumonia was much lower than that in infected mice (20 nM vs. 1000 nM). Meanwhile, mouse neutrophils produced more itaconate than human neutrophils after *M. pneumoniae* infection *in vitro*. This apparent lower capacity of human IRG1 to produce itaconate is in line with earlier reports for human macrophages(Michelucci 2013 PNAS, PMID: 23610393; Kohl 2023 EMBO Mol Med), and may be due to the reduced enzymatic activity as described previously(Chen et al, 2019, PNAS, PMID: 31548418).

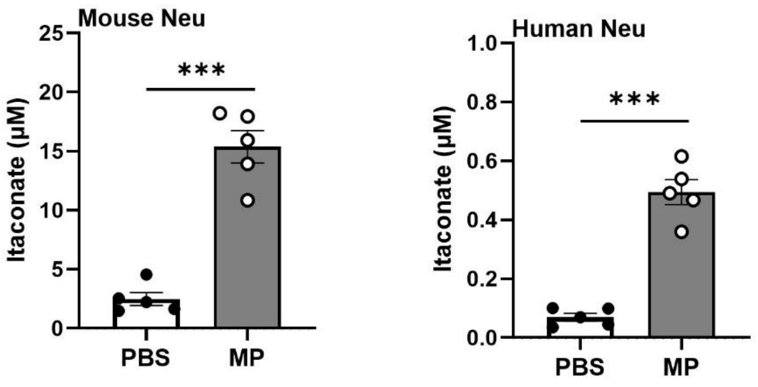

Figure 3: the approach to use adoptive transfer of wt and Acod1-/- neutrophils to corroborate the function of neutrophil-derived itaconate is valid. The results are consistent with the hypothesis, yet it is not demonstrated that the injected cells are indeed traveling to the lung or cause the effect on M. pneumoniae by a different mechanism. Therefore, the presence of transferred neutrophils in lung and BALF should be confirmed. This can be done using syngeneic markers like CD45.1/2. In addition, it should be tested whether itaconate in BLAF is indeed higher after transfer of wt neutrophils.

Response: Thanks. As you suggested, we confirmed the presence of a high level of transferred neutrophils by using CD45.1/2 recipient mice (S3 Fig). Moreover, we have supplemented itaconate concentration data. The result showed that the itaconate concentrations in BALF were higher after transfer of WT neutrophils than after transfer of *Irg1* KO neutrophils (Fig 3C).

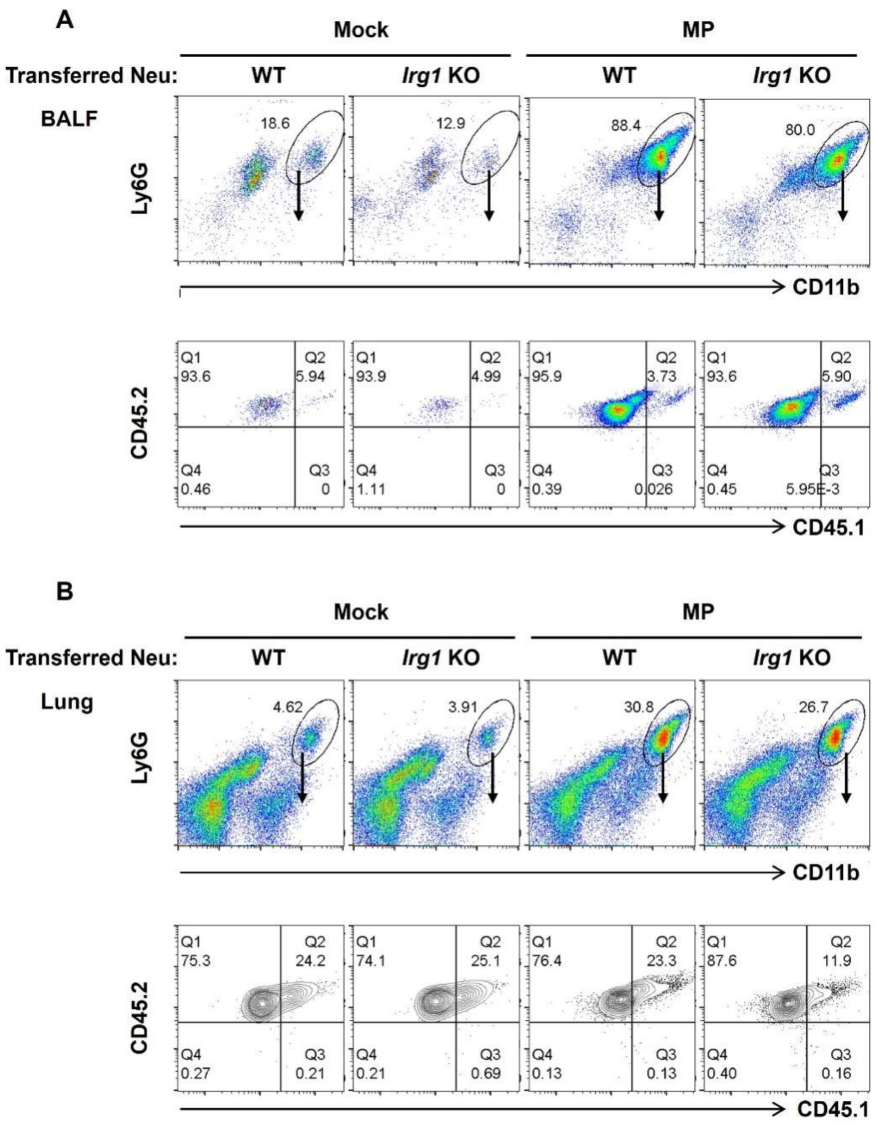

S3 Fig. Representative flow cytometric analysis of transferred neutrophils in the mouse lung and BALF. Sorted BM neutrophils (CD45.2 background) from WT and *Irg1* KO mice were intravenously transferred into CD45.1/2 mice, respectively. Then, the recipient mice were i.n. infected by M. pneumoniae (MP) or treated with PPLO medium (Mock group). Mice were sacrificed at day 1 after infection to analyze transferred neutrophils (CD45.1-CD45.2+ cells) and non-transferred neutrophils (CD45.1+CD45.2+ cells) in BALF (A) and lung (B) by flow cytometry.

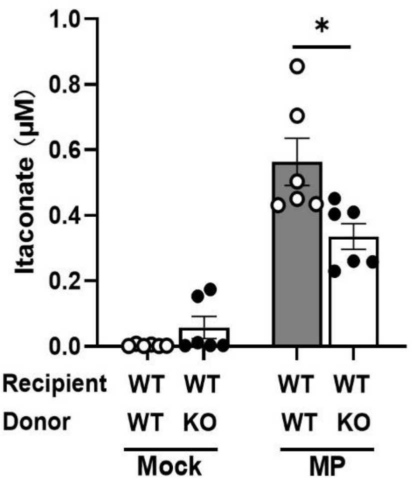

Fig 3C.

For the histopathology in Fig. 3F, the same criticism as in Fig. 1 applies. Please, provide quantitative data allowing statistical analysis.

Response: Quantitative data has been supplemented as follows.

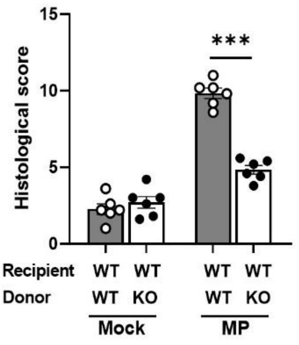

Figure 4: the treatment of infected mice with b-glucan has beneficial effects that are associated with reduced expression of Irg1 in BALF neutrophils and itaconate levels in the BALF. Whether this association reflects a causal link remains unclear because b-glucan can have many and diverse effects on the response to bacterial infection. E.g. the number of neutrophils recruited to the lung is very strongly reduced by b-glucan treatment which may be the major contributor to the effect on bacterial burden. To support the claim that b-glucan acts mainly as a IRG1 inhibitor, a direct reduction of IRG1 expression and itaconate production by neutrophils treated win vitro with b-glucan should be tested.

Response: We showed that β-glucan treatment significantly decreased the expression levels of *Irg1* mRNA and IRG1 protein in neutrophils induced by *M. pneumoniae* infection (S6A and S6B Fig). As suggested, we tested itaconate content in the culture supernatant, and the results showed that β-glucan treatment remarkably reduced itaconate production from infected neutrophils (S6C Fig), suggesting that β-glucan acts mainly as an inhibitor of IRG1 expression.

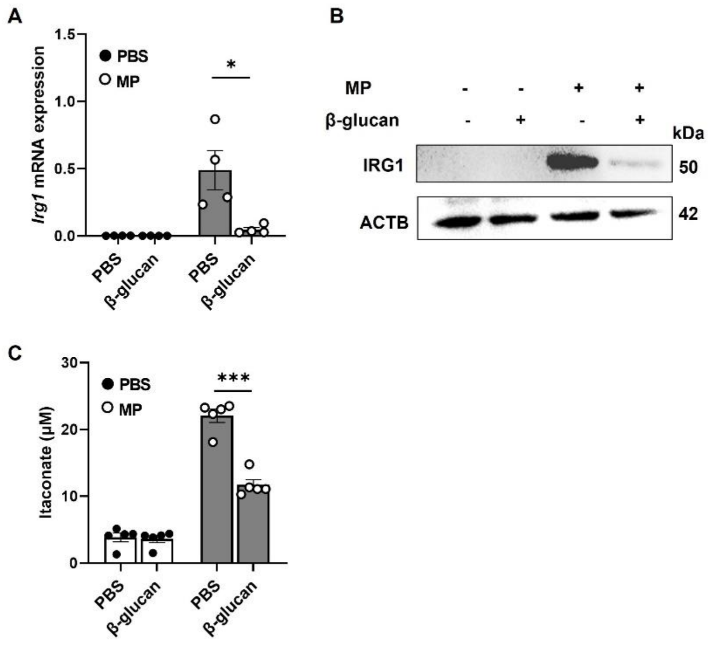

S6 Fig. β-glucan inhibits *M. pneumoniae*-induced *Irg1* expression in neutrophils *in vitro*.

Reviewer #2: None

Reviewer #3: 1. The patient cohort is poorly described. Basic characteristics are shown in Suppl Table 1, but nothing is mentioned about how severe disease is diagnosed: what about chest radiograph (CXR) findings and related CXR severity scores, hypoxemia requiring oxygen supply, and inflammatory parameters? Also it is unclear at what stage after onset of symptoms was BALF sampled for itaconate concentrations vs # neutrophils (Fig2J)? Also, findings shown in Fig 2J should be better discussed.

Response: Thank you. As you suggested, we have provided more information in S1 Table, including chest radiograph (CXR) findings and related CXR severity scores,

LDH levels, and the stage at which BALF was sampled. No patient requiring oxygen supply due to hypoxemia. Chest imaging score was evaluated according to the following criteria: non-consolidation, consolidation, consolidation and a small amount of pleural effusion, and consolidation and a medium to large amount of pleural effusion as described previously as described previously (Li et al., BMC Pulm Med. 2023, PMID: 37817172). Moreover, the findings shown in Fig. 2J was better discussed as shown above.

S1 Table. Demographic and clinical information of study patients with severe *M. pneumoniae* pneumonia.

Parameters	[a] Patients, n=17	[b] Patients, n=4
Age (mean ± SD）	6.05 ± 2.03	5.13 ± 1.92
Male (n, %)	7 (41.18)	3（75）
Female (n, %)	10 (58.82)	1（25）
Temperature (mean ± SD, ℃）	39.43 ± 0.79	39.1 ± 0.18
Respiratory rate (mean ± SD）	24.88 ± 2.71	25.25 ± 0.96
Day of cough (mean ± SD）	6.56 ± 3.46	3.5 ± 0.58
Day of fever (mean ± SD）	6.44 ± 3.44	3.75 ± 0.96
CRP (mean ± SD, mg/L）	24.51 ± 40.26	ND
HB (mean ± SD, g/L）	119.76 ± 7.38	114 ± 2.83
LDH (mean ± SD, U/L）	309.48 ± 111.52	ND
WBC (mean ± SD, x 109/L）	7.82 ± 3.56	6.22 ± 1.91
Blood neutrophils (mean ± SD, %）	64.96 ± 10.63	42.6 ± 23.99
Blood neutrophils (mean ± SD, x 109/L）	1.38 ± 0.73	2.55 ± 0.92
Blood lymphocyte (mean ± SD, %）	28.42 ± 14.43	46.95 ± 18.75
Blood platelet count (mean ± SD, %）	275.53 ± 86.95	443.5 ± 228.28
Chest CT findings		
Consolidation (n, %)	7 (41.17)	1 (25)
Bronchovascular bundles thickening (n, %)	12 (70.58)	0
Patchy high-density shadow (n, %)	17 (100)	4 (100)
Linear opacities (n, %)	5 (29.41)	0
Nodules (n, %)	2 (11.76)	0
Lymphadenopathy (n, %)	1 (5.88)	0
Pleural effusion (n)	0	0
Chest imaging score		
1 (n, %)	10 (58.82)	1 (25)
2 (n, %)	7 (41.18)	3 (75)

[a] BALF samples were used for measuring itaconate concentrations, corresponding to Fig 2J.
[b] BALF samples were used for determining IRG1 expression in neutrophils and non-neutrophils by flow cytometry, corresponding to Fig 2K.
CRP: C-reactive protein; HB: Hemoglobin; WBC: White blood cell; SD: Standard deviation; ND: not determined.

2. Adoptive transfer of Igr1-KO neutrophils attenuates MPP: read out is done on day 3 p.i.

-Are effects still effective at later stages in the disease?

-is attenuated MPP also reflected in less weight loss in mice?

-how many neutrophils were transferred?

Would have expected a few neutrophils in lungs from control mice, especially after iv injection.

Response: Thanks for the questions. As we focus on the role of itaconate-producing neutrophils during *M. pneumoniae* infection, the lung inflammation was assessed after 3 days postinfection when itaconate levels and neutrophil numbers were high and lung inflammation was pronounced. As a number of studies have shown that myeloid-derived itaconate suppresses CD8+ T-cell responses in cancer (Zhao et al. 2022, Nat Metab. PMID: 36376563; Chen et al. 2023, Sci Adv. PMID: 37115931) and infection (Ramalho et al, 2024, Cell Metab. PMID: 38325373), we thus speculate that neutrophil-derived itaconate would be still effective at later stages of *M. pneumoniae* infection. We will carry out relevant investigation in the near future. As described in Methods and Materials, we transferred 3 million neutrophils into recipient mice. We did not observe any difference in the weight loss between mice receiving WT neutrophils and mice receiving *Irg1* KO neutrophils after *M. pneumoniae* infection, although infection did cause a slight decrease in mouse weight (S4 Fig). Moreover, we confirmed the presence of a high level of transferred neutrophils by using CD45.1/2 recipient mice as shown above (S3 Fig).

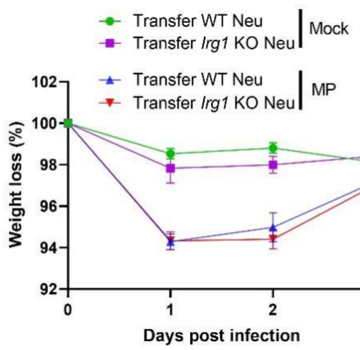

S4 Fig.

3. Did the authors not expect that suppletion with exogenous ITA would result in more severe inflammation/pathology in the lungs (Fig 1)?

Response: Yes. We initially expected that suppletion with exogenous itaconate would lead to more severe lung inflammation/pathology in mice after *M. pneumoniae* infection. However, we did not observe such effects. This suggests that the endogenous itaconate production induced by *M. pneumoniae* infection may be sufficient to cause the effects. We have added this information to the Discussion section.

Part III – Minor Issues: Editorial and Data Presentation Modifications

Please use this section for editorial suggestions as well as relatively minor modifications of existing data that would enhance clarity.

Reviewer #1: Figure 6: the header for this part in the Results section reads "Itaconate inhibits neutrophil survival" which is the opposite of what is shown in the figure.

Response: Thanks. We have revised the header as "Itaconate inhibits neutrophil apoptosis during *M. pneumoniae* infection".

Figure 7: the use of knockout neutrophils instead of the chemical inhibitors for TLR2/4 and Myd88. It is not stated whether the phosphorylation of STAT1 refers to tyrosine or serine.

Response: We used chemical inhibitors of TLR2/4 and MyD88 to investigate whether

these molecules are involved in upregulating *Irg1* expression after *M. pneumoniae* infection. The phosphorylation of STAT1 refers to tyrosine and we have added this information in Western blotting part in the Materials and Methods section.

Reviewer #2: There are some minor weaknesses/revisions needed:

1) Line 256, heading: States "Itaconate inhibits neutrophil survival during M. pneumoniae infection", but the data shows that itaconate inhibits apoptosis. Thus, the header needs to be revised.

Response: Thanks. We have revised the header as "Itaconate inhibits neutrophil apoptosis during *M. pneumoniae* infection".

2) Make sure that all abbreviations in figures are defined in figure legends. For example, Figure 2D has the abbreviation "Lung leukocytes-Neu". I initially interpreted this as designating only lung neutrophils, but it actually refers to lung leukocytes depleted of neutrophils. This should be clarified in the figure legend. Please review the other figures.

Response: "Lung leukocytes-Neu" in Fig 2D has been clarified in the figure legend. Lung leukocytes-Neu: Lung leukocytes depleted of neutrophils. Non-Neu: CD45+CD15-CD16- cells in Fig 2K.

3) Format of references need to be consistent. There are several references where every first letter of word is capitalized, while others do not.

Response: We have revised the format of all references according to the requirement of *PLOS Pathogens*.

4) Need to italicize all species names in references, e.g. Mycoplasma pneumoniae, Staphylococcus aureus, etc.

Response: All species names in references have been italicized.

Reviewer #3: 1. Fig 5C: how is % SDH activity calculated for each group? I would have expected that MP infection would induce SDH activation and thus result in

higher levels compared to DMSO-PBS group.

Response: SDH activity in neutrophils was determined by using a MitoCheck® complex II activity assay kit (Cayman, USA, 700940) according to the manufacturer's instructions. We first plotted data as absorbance (y-axis) vs time (x-axis), and calculated the slope for the linear portion of the curve to determine the reaction rate, followed by calculating SDH activity (%) using (Rate of Sample wells/Rate of Control wells) ×100. We observed itaconate inhibited SDH activity in neutrophils; however, *M. pneumoniae* infection within a short time did not impact SDH activity in neutrophils (Fig. 5C). Our results suggest *M. pneumoniae* infection within 2 h did not influence SDH activity in neutrophils. We have added the calculation methods in Materials and Methods section.

2. lines 107-112: describe what tissue is analysed

Response: We have described the specific sample information as follows: Compared with mock infection, the mRNA expression of *Irg1* gene in lung tissue was significantly increased at day 1 postinfection (p.i.) and day 3 p.i., returning to almost baseline levels at day 7 p.i. (Fig 1A). Meanwhile, the IRG1 protein expression in lung tissue by western blot displayed a similar change (Fig 1B). Consistent with IRG1 expression, the itaconate levels in BALF were dramatically elevated at day 1, especially at day 3 p.i. and returned to baseline levels at day 7 p.i. (Fig 1C).

Line 115: typo: "with intranasally administrated with "

Response: We have changed to "with intranasal administration with".

Line 116: lower level of MP load > should be "lower load of MP bacteria"

Response: We have changed to "lower load of *M. pneumoniae* bacteria".

line 117: what is a "pure MP infection"?

Response: Pure *M. pneumoniae* infection refers to intranasally administrated only with *M. pneumoniae*. To make it clear, we have deleted "pure".

line 392: "regents" > reagents

Response: We have changed to “reagents”.

Thanks again for your time and valuable suggestions.

Sincerely,

Aihua Lei, Ph.D.
Institute of Pathogenic Biology, University of South China
leiaihua18@usc.edu.cn
