# Supplementary material for: Suppressing neutrophil itaconate production attenuates Mycoplasma pneumoniae pneumonia
Source: PLoS Pathog. 2024 Nov 5;20(11):e1012614. doi: 10.1371/journal.ppat.1012614 (PMC11567624; doi:10.1371/journal.ppat.1012614)
Supplement: Attachment — Submitted filename: Our response to comments.docx [file ppat.1012614.s015.docx]

**PPATHOGENS-D-24-00902R1**

Response to Editors and Reviewers

Dear Editors and Reviewers,

We would like to thank the editors and all the reviewers for their valuable comments on our manuscript. Following the comments, we have made appropriate changes to our manuscript (modifications are marked in red). Hopefully, you will find our revised manuscript suitable for publication at *PLOS Pathogens*. Our point-to-point responses to the editors’ and reviewers’ comments are described below:

**Response to the Editors**

The revised version of the paper is much improved, but reviewer #3 has some small points to address. In particular, the first point requires a small amount of explanation, and the third warrants some clarification. Once these minor issues are satisfactorily addressed, I see no barrier to publication.

Response: We greatly appreciate your good comments to our work. We have answered all of Reviewer #3’s questions as shown below. Moreover, we have checked all the references and replaced incorrect reference 2 (PMID: 5816304) with a correct one (PMID: 39117175). No cited papers have been retracted. Meanwhile, we have improved Materials and Methods section and supplemented “Extraction of *M. pneumoniae* LAMP”. We have checked all the figures and the numerical data used in the figures are included in S1 Data and S2 Data.

**Responses to Reviewer's Comments**

**Part I - Summary**

Please use this section to discuss strengths/weaknesses of study, novelty/significance, general execution and scholarship.

Reviewer #1: The authors have addressed all my comments and provided additional data that support their conclusions.

They even allowed me to contribute to the Discussion section by copy/pasting my comment on mouse/human differences related to Figure 2. Thus, I am more than satisfied.

Response: Thanks for your valuable comments.

Reviewer #2: The authors appear to have address the concerns raised by the reviewers. They have addressed my comments well. It is a very interesting paper which may have identified a mechanism used by *M. pneumoniae* to persist along the respiratory tract, despite an intense inflammatory response.

Response: Thanks for your good comments.

Reviewer #3: The authors have adequately answered most of the questions; unfortunately a few items yet remain to be better explained:

1. Fig S3 showing flowcytometry plots of neutrophils in lung and BALF cells to indicate the presence of transferred donor neutrophils in these tissues. Hereto the authors used CD45 congenic mice.

Why were CD45.1/.2 chosen as recipient? how can the transferred CD45.1 donor cells be distinguished? Ideally CD45.1 are transferred into CD45.2 recipients, leaving two single positive populations in the CD45.1 vs CD45.2 plots.

What antibodies were used to stain for CD45.1 and CD45.2 (not present in methods).

Response: Thanks for the questions. We used CD45.1/.2 mice because we did not have enough CD45.1 mice. To confirm the presence of transferred donor neutrophils in the mouse lung upon *M. pneumoniae* infection, bone marrow neutrophils from WT (CD45.2 background) and *Irg1* KO (CD45.2 background) mice were intravenously transferred into CD45.1/2 mice, respectively. Thus, CD45.1^-^CD45.2^+^ neutrophils in BALF and lung of recipient mouse are from transferred donor cells while CD45.1^+^CD45.2^+^ neutrophils are from recipients. We used PE anti-mouse CD45.1 (Invitrogen, 12-0453-91) and APC anti-mouse CD45.2 (BD Biosciences, 561875) to stain CD45.1 and CD45.2. We have supplemented antibody information in methods.

2. The answer to whether itaconate may be effective at later stages in infection is not correct in my opinion: the authors cite studies demonstrating effects on CD8 T cell responses. however, ITA affects the priming of T cells (so in early stages) leaving "crappy CD8 effector T cells" that cannot resolve the infection at the later stages. So you could argue it's a matter of semantics, as the crappy effector T cells are an effect of ITA. however in the Discussion, lines 398-400, the authors phrased it better.

Response: We agree with your comment.

3. Regarding the suppletion of exogenous ITA in wt mice and tissue damage: apparently the effects of ITA are not dose-dependent; its presence as such may be enough to evoke damage, or there may be a certain threshold? this should be better worded in the Discussion than is done now: lines 381-384: The fact that supplementation with exogenous itaconate did not lead to more severe lung inflammation in WT mice suggests that the endogenous itaconate production induced by *M. pneumoniae* may be sufficient to cause the effects.

Response: Thanks. As for the suppletion of exogenous itaconate in WT mice and lung inflammation, we have revised our discussion as: Notably, we found that supplementation with exogenous itaconate did not lead to more severe lung inflammation in WT mice, suggesting that the effects of itaconate may not be dose-dependent or there may be a certain threshold. This indicates that the endogenous itaconate production induced by *M. pneumoniae* infection may be sufficient to evoke lung damage.

Minor:

-MFI=mean fluorescenCE intensity, not fluorescent.

-in methods "Mycoplasma pneumoniae and infection: add "mouse model"

Response: Done.

**Part II – Major Issues: Key Experiments Required for Acceptance**

Reviewer #1: (No Response)

Reviewer #2: None

Reviewer #3: (No Response)

**Part III – Minor Issues: Editorial and Data Presentation Modifications**

Reviewer #1: (No Response)

Reviewer #2: No major concerns.

Reviewer #3: (No Response)

Thanks for your time and valuable suggestions.

Sincerely,

Aihua Lei, Ph.D.

Institute of Pathogenic Biology, University of South China

[leiaihua18@usc.edu.cn](mailto:leiaihua18@usc.edu.cn)
